# Randomised, Single Blind, Controlled, Three-Month Clinical Trial on the Evaluation and Treatment of the Ocular Surface Damage Following Phacoemulsification

**DOI:** 10.3390/vision6030042

**Published:** 2022-07-06

**Authors:** Gemma Caterina Maria Rossi, Carmine Tinelli, Giovanni Milano, Sara Lanteri, Gabriella Ricciarelli, Laura Giannì, Gian Maria Pasinetti, Luigia Scudeller

**Affiliations:** 1 Department of Surgical Sciences, University Eye Clinic, Fondazione IRCCS Policlinico San Matteo, 27100 Pavia, Italy; giovanni.milano@unipv.it (G.M.); lanteri.sara@gmail.com (S.L.); gabriella.ricciardelli@gmail.com (G.R.); lauragianni@gmail.com (L.G.); 2 ASST Bergamo Est, Ospedale Locatelli, 24020 Piario, Italy; 3 Clinical Epidemiology and Biometric Unit, Scientific Direction, Fondazione IRCCS Policlinico San Matteo, 27100 Pavia, Italy; ctinelli@gmail.com (C.T.); l.scudeller@smatteo.pv.it (L.S.); 4 Medicine and Surgery Faculty, University of Pavia, 27100 Pavia, Italy; 5 Eye Unit, Istituto Beato Palazzolo, 24100 Bergamo, Italy; gianmaria.pasinetti.md@gmail.com

**Keywords:** cataract surgery, phacoemulsification, dry eye, BUT, corneal staining, Schirmer test, National Eye Institute Visual Function Questionnaire (NEI-VFQ), Ocular Surface Disease Index (OSDI), sodium hyaluronate, carboxymethylcellulose

## Abstract

Background: To determine efficacy of two lacrimal substitutes on signs and symptoms of ocular surface disease after phacoemulsification; to determine impact of surgery on patients’ vision related quality of life. Monocentric, randomised, physician blinded, three parallel groups clinical trial. Design and Methods: Patients in the operative list for phacoemulsification have been screened for eligibility; they underwent (at time 0, 15, 45 and 90 days): slit lamp examination; tear film break-up time (BUT); corneal staining; tear volume; 25-item National Eye Institute Visual Function Questionnaire (NEI-VFQ); Ocular Surface Disease Index (OSDI). Treatments to be compared were: 1. standard of care-SOC (lomefloxacine and tobramicine/dexamethasone fixed combination 4 times a day for 2 weeks), 2. SOC + carboxymethylcellulose sodium 0.5% and glycerin 0.9%, 3. SOC + Sodium Hyaluronate 0.15%. Study treatment started at T15. Groups were compared with parametric or nonparametric tests, and with Pearson’s χ^2^ test. Correlation between continuous variables was assessed by means of Pearson’s or Spearman’s coefficient. Results: Fifty-three patients were enrolled. At 45 and at 90 days from surgery, the group receiving lacrimal substitutes presented better BUT and Schirmer I test (*p* = 0.009, <0.001, <0.001 and 0.001, respectively); dry eye presence showed significant difference by group at time 90 (*p* = 0.019). General vision, near activity and vision-specific dependency subscales improved after surgery (*p* = <0.001, 0.004 and 0.048, respectively). At 45 and 90 days from surgery, the OSDI score significantly changed (*p* < 0.001).Conclusions: Cataract surgery causes the onset or the worsening of dry eye. Use of artificial tears can significantly reduce symptoms and signs of dry eye in patients after phacoemulsification.

## 1. Introduction

Cataract is the most common disease leading to blindness worldwide. At the same time, cataract surgery is the most classic and successful surgery in the field of ophthalmology.

Cataract surgery has given innumerable patients good visual acuity, but dry eye-associated symptoms, such as foreign body sensation, fatigue, redness and watery eyes, frequently occur after the procedure [1,2,3]. These symptoms may be accompanied by signs, such as superficial punctatae keratitis and epithelial defects on the cornea.

Some studies have reported aggravation of dry eye symptoms and signs after cataract surgery, both in patients with pre-existing dry eye and in subjects without ocular surface disease [1,2,4,5].

Many factors may affect the ocular surface status after cataract surgery [2]. Eye drops containing preservatives, such as benzalkonium chloride, and topical anaesthesia are well known to have toxic effects on the corneal epithelium [6,7]. The postoperative dry eye may also be associated with exposure to light from the operating microscope and to incision shape [5].

In cataract surgery, although the width of the incision is very small, the incision depth extends to involve the entire cornea and to damage the corneal innervation and therefore tear production [8].

Ocular surface disease may be diagnosed by objective methods, such as the tear film break-up time (BUT), Schirmer’s test and fluorescein staining tests [9].

The onset of an alteration in the health of the ocular surface, particularly symptoms of discomfort, may impact patients’ daily life, representing an important aspect to consider in the follow-up. To evaluate the impact of the surgery itself and of the ocular surface disease on patients’ quality of life, there are dedicated questionnaires, such as the 25-item National Eye Institute Visual Function Questionnaire [7] and the Ocular Surface Disease Index (OSDI) [10].

Data from the literature suggest that eye drops should be carefully administered after cataract surgery to avoid or reduce the incidence of dry eye after cataract surgery; however, to our knowledge, few papers have addressed the issue of reduction of dry eye symptoms in patients undergoing cataract surgery [11,12,13,14].

The principal objective of the study was to determine whether the use of eye drops for dry eye (carboxymethylcellulose sodium 0.5% or sodium hyaluronate 0.15%) are better than standard of care (SOC) in improving signs (BUT, Schirmer’s test, punctatae keratitis) and symptoms of ocular surface disease (OSDI). An additional objective of the study was to determine impact of surgery on patients’ vision-related quality of life (NEI-VFQ 25).

## 2. Methods

A monocentric, randomised clinical trial, physician blinded, with three parallel groups (standard of care and two treatment arms) was approved by the Ethical Committee of the Fondazione (Proc. N. 20090038495).

All patients in the operative list for phacoemulsification at the Eye Clinic of IRCCS Policlinico San Matteo Foundation were screened for eligibility at the routine pre-surgery visit (T0).

To be eligible, the patients needed to give their written informed consent and to undergo cataract surgery. Even in the case of bilateral involvement, each patient was included once, at the time of first surgery. All enrolled patients received an explanatory letter directed to their general practitioner.

Patients with any of the following conditions were excluded: pre-existing ocular surface pathology or chronic therapy in the same eye; pre-existing chronic use of artificial tears in the same eye; prior ocular or adnexa surgery (i.e., vitreoretinal or anti-glaucoma surgery) in the same eye; abnormality of the nasolacrimal drainage apparatus in the same eye; systemic disease related to dry eye (e.g., rheumatoid arthritis, rosacea); blepharitis in the same eye; ocular allergy.

All patients underwent the following examinations and tests: slit lamp examination; tear film break-up time (tf-BUT) using fluorescein; corneal staining with fluorescein; tear volume (Schirmer I test); 25-item National Eye Institute Visual Function Questionnaire (NEI-VFQ); and Ocular Surface Disease Index (OSDI). Dry eye was defined as the concomitant presence of corneal staining >0 and BUT <10 s.

The treatments to be compared were 3.

1. Standard of care. The SOC treatment in use at the Clinica Oculistica was as follows: from the day of surgery, the following eye drops were used: lomefloxacine and tobramicine + dexamethasone fixed combination 4 times a day for 2 weeks; then Tobramicine + dexamethasone fixed combination 2 times a day until day 25 from surgery.
2. Arm A: patients received an artificial tear designed with a dual action formula which contains carboxymethylcellulose 0.5% (a long lasting lubricant), glycerin (an ideal moisturiser), and purite (a safe gentle preservative) (Optive ^®^). This blend of ingredients can provide long-lasting, deep hydration of the tear film and corneal cells, as well as an important osmoprotection. The eye drop is safely drawn into the corneal epithelial cells below the surface to osmotically protect against hypertonic stress. That’s why it is recommended for patients with intermittent dry eye symptoms.
3. Arm B: patients received a sterile isotonic ophthalmic solution made up of hyaluronic acid 0.15%, sodium hydroxymethylglycinate (N-IG) and 4 different amino acids (L-proline, L-glycine, hydrochloride L-lysine and L-leucine) (BluYalA ^®^). It is proposed to be able to supplement insufficient tear secretion and restore the physiological conditions of the tear film thanks to: hyaluronic acid, which stays on the ocular surface for a long time and stabilises the tear film for its mucoadhesive characteristics; the N-IG, a new generation amino acid derivative with an antimicrobial activity that should have an effective preserving action and no negative effects on the surface of the eye; and the amino acids L-proline, L-glycine, hydrochloride L-lysine and L-leucine, which are important in maintaining structural condition and metabolic processes of the cornea. Actually, the product is used to protect, humidify and lubricate the surface of the eye bringing particularly extended relief to disturbances caused by mechanical, environmental and visual stress.

At enrolment, patients were randomised, according to a computer-generated randomisation list, to any of the three groups, but study treatment (arm A and arm B) started at T15 (i.e., 15 days from cataract surgery) with 1 drop 3 times a day into the operated eye until T90 (90 days from surgery).

Lomefloxacine was instilled 5 times a day, starting from the day prior to surgical procedure. On surgery day (Tx), eye drops with tropicamide 1% were used 3 times over half an hour to dilate the pupil. Topical surface anesthesia (benoxinate) was administered before surgery.

**Figure 1:**
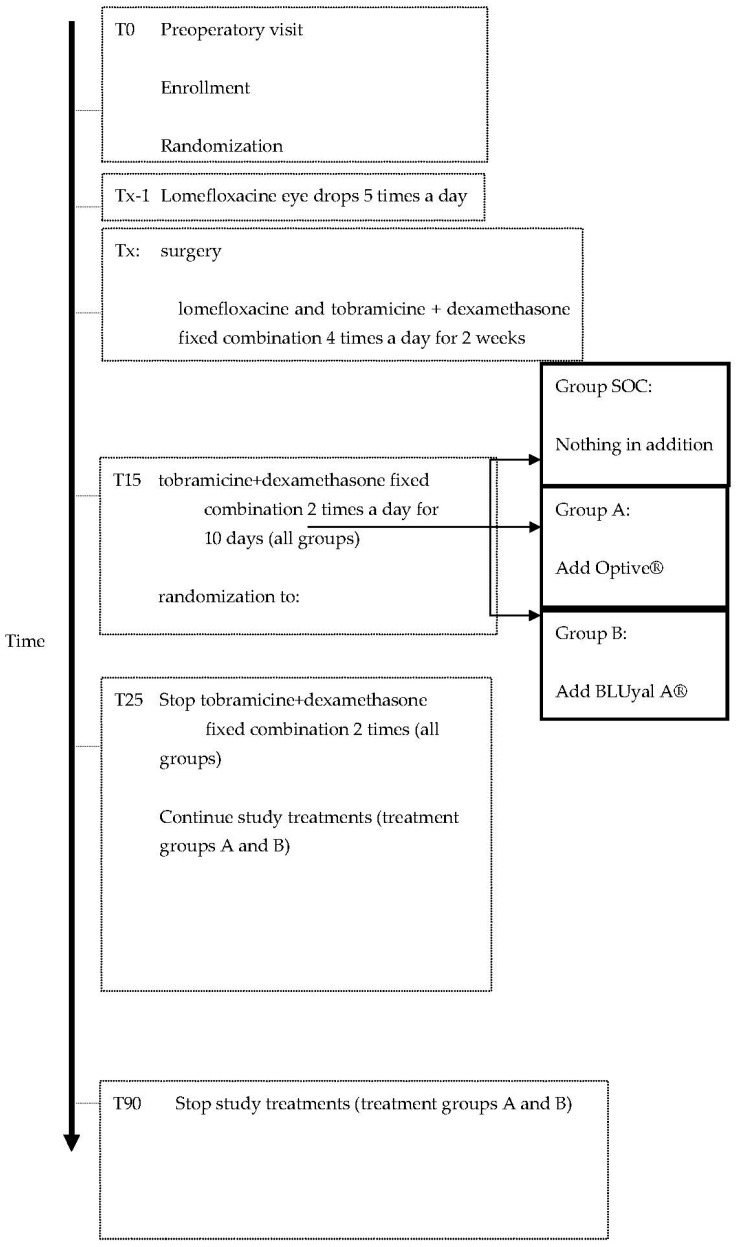
Flow-chart of the study design.

Figure 1 reports the treatment flow-chart.

Of note, all the patients were operated by the same surgeon (Alessandro Bianchi), to avoid inter-operator variability.

AMO Sovereign was used for cataract surgery: a chop phacoemulsification technique with a 2.75 mm-sized corneal incision was performed using a precalibrated blade at the superior location. A 1.0 mm-sized paracentesis was made about 50° apart from the main incision. A foldable acrylic PC-IOL was implanted in the capsular bag, after irrigation and aspiration, hydrosuture was performed. An assistant hydrated the cornea upon the surgeon command or when the assistant felt like hydration was necessary. Postoperatively, the eye was covered for 1 day.

### 2.1. Adverse Events

Even after discharge, patients were advised to promptly report to the study medical doctors in case of any adverse event and, upon discharge, they were provided with the relevant contact information. Adverse events were anticipated to be few. The possible adverse events to study products were low patient’s tolerability.

Adverse events related to surgery or to the standard products were treated according to the common practice at the eye clinic and recorded on the case report form in the standard way.

### 2.2. Blinding/Masking

Study medications were dispensed by dedicated personnel (LG), who did not further participate in the study. Ophthalmologists performing ocular surface evaluation were blinded to study treatment assignment of the patient (GCMR, SL, GR): all study assessments were performed twice by two different operators.

Due to resource restraints, it was impossible to blind the patients to study treatments; however, patient’s subjective measures of efficacy were not the main study objective.

### 2.3. Randomisation Procedures

A randomisation list 1:1:1 was generated and maintained at the Direzione Scientifica of the Fondazione. However, the two treatment groups were considered together in subsequent analyses. Following inclusion in the study of a patient, the study investigator alerted via e-mail the contact person of the Direzione Scientifica, who assigned the patient to one of the treatment arms following the randomisation list. This information was provided only to the person in charge of product supply at the Clinica Oculistica.

All patients underwent 6 visits at T0, Tx, T1, T15, T45, T90 from surgery, according to the timetable reported as Table 1.

### 2.4. Ocular Examination

#### 2.4.1. Schirmer I Test (ST-I)

The ST-I was evaluated without corneal anesthesia. The test lasts 5 min, and the length of wetted paper is directly read off the scale on the paper itself. The ST-I was only performed once.

#### 2.4.2. Tear Film Break-Up Time (tf-BUT)

The time interval between ST-I and tf-BUT was at least 10 min. A fluorescein-impregnated strip wet was placed in the inferior fornix and the patient was asked to blink several times. The tf-BUT was evaluated measuring the interval between a complete blink and the appearance of the first area of tear film break-up on the corneal tear film, using a cobalt blue filter on the slit lamp microscope. The average value of three measurements has been calculated. The tf-BUT was performed by two observers, and the mean value was used. Also, tf-BUT values greater than or equal to 10 s were coded as normal and tf-BUT values less than 10 s as abnormal.

#### 2.4.3. Fluorescein Staining for the Evaluation of the Corneal Damage

The presence of corneal staining was defined as more than one dot of fluorescein staining over the corneal surface. Superficial punctate keratitis has been graded in according to the corneal fluorescein staining scale by determining the area and density of the lesion on a 0–3 scale, where 0 is absence of keratitis punctata, 1 = Mild (a few punctatae of staining but less than 10% coverage of the corneal surface; 2 = Moderate (10–50% coverage of the corneal surface); and 3 = Severe (more than 50% coverage of the corneal surface) [15]. For the statistical analyses, the corneal staining was evaluated as absent (grade = 0) or present (grade > 0).

#### 2.4.4. Conjunctival Hyperaemia

Conjunctival hyperaemia was evaluated according to a grading scale ranging from 0 to 2 points: grade 0 = none, grade 1 = mild/moderate injection, grade 2 = severe injection [15]. For the statistical analyses, the conjunctival injection was evaluated as absent (grade = 0) or present (grade > 0).

#### 2.4.5. Ocular Surface Disease Index (OSDI)

The OSDI is a disease-specific questionnaire used to quantify the specific impact of dry eye on vision-related quality of life. It includes three subscales: ocular discomfort (OSDI-symptoms), functioning (OSDI-function) and environmental triggers (OSDI-triggers) [10]. The questions refer to a one-week recall period, and possible responses refer to the frequency of the disturbance. OSDI subscale scores can range from 0 to 100, with higher scores indicating more problems or symptoms. The OSD index (OSDI) was considered in the study to evaluate the impact of dry eye with the following scores: 0–12 normal, 13–22 mild, 23–32 moderate and 33–100 severe symptoms.

#### 2.4.6. NEI-VFQ 25

The patients’ quality of life was examined with the Italian version of the 25-item National Eye Institute Visual Function Questionnaire. The 25 item National Eye Institute Visual Function Questionnaire [9] is a non-disease-specific instrument designed to measure the impact of some ocular disorders on vision-related quality of life. Depending on the item, responses to this questionnaire pertain to the frequency or severity of a symptom or a problem with the functioning. The NEI-VFQ scores can range from 0 to 100 with lower scores indicating more problems or symptoms.

### 2.5. Statistics

The sample size calculations were based on the following assumptions: mean tf-BUT at t45 = 6 in the control group and = 10 in each of the treatment groups, common standard deviation 4, power 80%, alpha error 5%.

To assess these effects by means of one-way ANOVA, sample sizes of 16 (control group), 16 and 16 in each of the treatment groups were required.

Descriptive statistics were produced for demographic, clinical and laboratory characteristics of cases. Mean and standard deviation (SD) are presented for normally distributed variables, and median and interquartile range (IQR) for non-normally distributed variables, number and percentages for categorical variables. Groups were compared with parametric or nonparametric tests, according to data distribution, for continuous variables and with Pearson’s χ^2^ test (Fisher exact test where appropriate) for categorical variables. Correlation between continuous variables was assessed by means of Pearson’s or Spearman’s coefficient, according to data distribution.

In all cases, 2-tailed tests were used. *p*-value significance cut-off was 0.05.

To assess changes over time of clinical parameters, regression methods for repeated measures within patients (and eye) were applied.

Stata computer software version 14.0 (Stata Corporation, 4905 Lakeway Drive, College Station, TX, USA) was used for statistical analysis.

## 3. Results

Fifty-three eyes of 53 patients were included, and all patients completed the study. Neither adverse events related to added treatments nor complicated surgery were recorded.

The median age was 74.6 [68.4–78.7]; 34 were female (64.1%). Sixteen patients were randomised to SOC and 37 received a lacrimal substitute at 15 days visit.

### 3.1. Signs

As expected in a randomised trial, at baseline, there were no significant differences between groups: the visual acuity was generally low (0.3–0.4 decimals); tf-BUT, corneal surface status (conjunctival hyperaemia and corneal staining) and Schirmer Test I were mainly within normal ranges, and dry eye was present in 20 (37.7%) patients.

Table 2 shows all clinical data by group.

Fifteen days after surgery, the visual acuity significantly improved in both group and it was maintained high for all the studied period, changing from 0.3–0.4 decimals at baseline to 0.9–1.0 decimals at t90 (*p* < 0.001).

**Figure 2:**
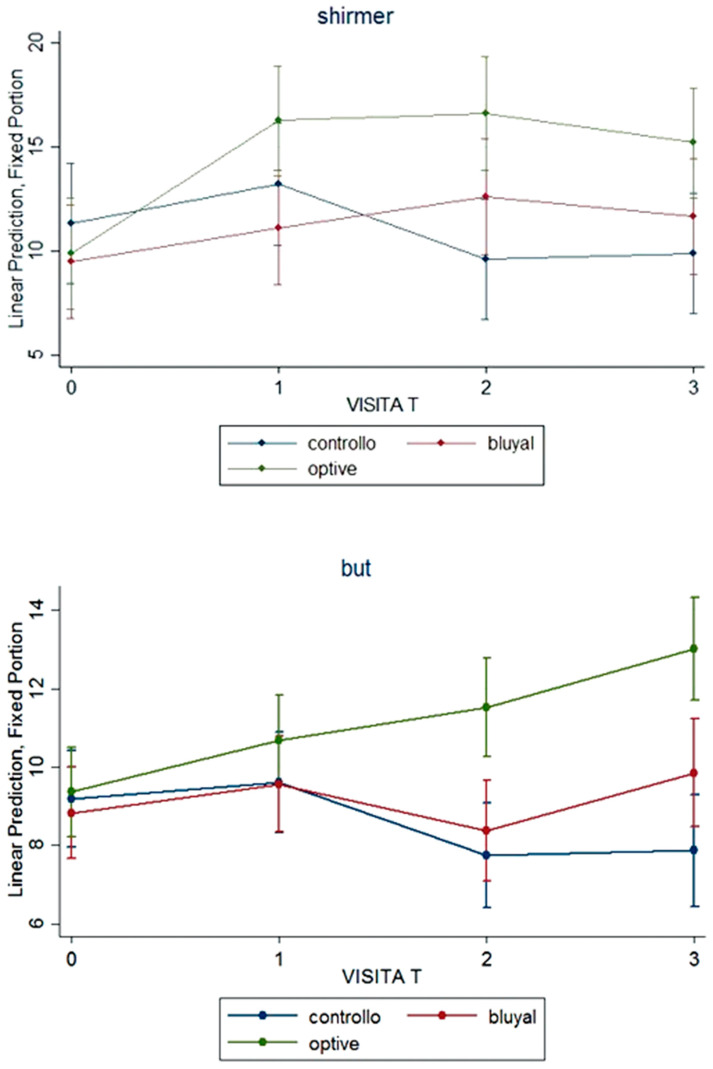
Signs of dry eye: Shirmer test and break-up time (BUT) over the time in the three groups.

Both at 45 and at 90 days from surgery, the group receiving lacrimal substitutes presented a statistically significant better tf-BUT and Schirmer I test (*p* = 0.009, <0.001, <0.001 and 0.001, respectively); dry eye presence changed over the time with a significant difference by group at time 90 (*p* = 0.019) (Figure 2). In detail, Schirmer test was unchanged over time (from 10 [10–12] mm to 10 [7–11.5] mm) in the SOC group, while slightly improved (from 10 [7–10] mm to 12.5 [10–17] mm) in patients receiving lacrimal substitutes (*p* = 0.001). Tf-BUT slightly worsened in the SOC group but improved in treated group (*p* < 0.001. Dry eye was present in a high percentage of patients receiving the SOC therapy (50%), but only in 25% of those treated with SOC plus an artificial tear (*p* = 0.019). The presence of dry eye syndrome significantly reduced from baseline to 90 days visit from 40% to 25% (*p* = 0.003). Corneal fluorescein staining was infrequent (and only of mild grade) and did not change over the time, such as conjunctival hyperaemia.

### 3.2. Symptoms

Vision-related quality of life examined with NEI-VFQ 25 revealed a good quality of life with an high total mean score at baseline that slightly improved after surgery. General vision (GV), near activity (NA) and vision-specific dependency (VSD) subscales improved after cataract extraction with a significant difference by group (*p* = <0.001, 0.004 and 0.048, respectively) (Table 3).

Regarding ocular surface-related quality of life, at baseline, both groups revealed a mild OSDI score, but at 45 and 90 days from surgery, the score statistically improved to “normal” in patients receiving a lacrimal substitute, while it changed to “moderate” in the SOC group (Table 2), with a statistically significant difference between groups (*p* < 0.001).

**Figure 3:**
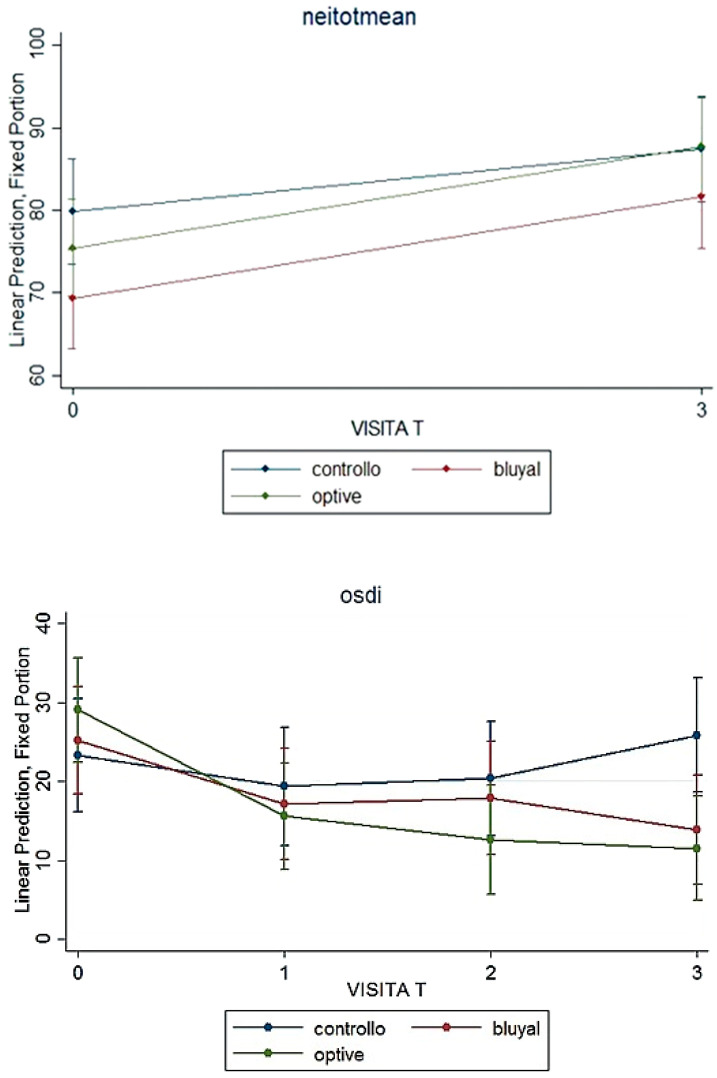
Symptoms and quality of life: NEI-VFQ total mean scoe and OSDI over the time in the three groups.

Figure 3 reports data about both questionnaires during the follow-up.

## 4. Conclusions

The present study found that dry eye appears 15 days after cataract surgery leading to signs and symptoms that adversely affect the patient’s well-being, our data shows that the use of artificial tears reduces both signs and symptoms of dry eyes, immediately improving patient satisfaction and therefore, consequently, also the outcome of the surgery.

Dry eye is a multifactorial disease of the ocular surface representing the most common disorder of the eye with a prevalence ranging from 5% to 35% in the population aged 50 years and older. The variation in observed prevalence relates to differences in the definition of disease used [16]. In our patients DES was present in 37.7% of subjects at baseline.

“Post-surgery dry eye” has a transient nature explainable by the fact that the inflammatory cytokines also induce the synthesis of a number of neurotrophic factors, stimulating the regeneration of corneal nerves in few weeks [17].

Dry eye after cataract surgery is related to numerous causes mainly including ocular epithelial injury due to corneal exposure and nerve cell injury during surgery [18,19]. To avoid an operator-dependent influence on surgery, the same surgeon operated on all the patients, paying particular attention to the ocular surface status during all the phases of the phacoemulsification and adopting every measure to reduce the injury to corneal nerves, as described in the Methods section. The result was that 15 days after cataract extraction and, therefore, after a 4 times a day steroid and antibiotic eye drop use, DES was present in 43.1% of patients.

According to previous literature [11,12,13,14], the prescription of artificial tears significantly improved both signs and symptoms of dry eye in our patients reducing the percentage of dry eye presence to 25%. Our data recorded some differences between the two lacrimal substitutes examined, but the sample did size not allow the direct comparison between the lubricant treatment groups.

Corneal incisions made during cataract surgery can release inflammatory mediators (HLA-DR and CD3) similar to the inflammation that appears with dry eye alone [11]: the use of topical steroids combined with the use of artificial tears reduces the expression of inflammatory markers; moreover, artificial tears wash away inflammatory mediators, improving lacrimal clearance. The reduction of inflammation improves the ocular surface status, leading to a decrease in dry eye symptoms too.

Dry eye, in fact, generates tear film instability that may affect quality of vision and may cause visual fluctuation to put in a differential diagnosis with other more severe surgical complications, such as corneal oedema, residual astigmatism, refractive error, cystoid macular oedema. The onset of ocular surface anomalies due to dry eye after cataract extraction negatively impacts vision-related quality of life as demonstrated by our findings. Therefore, cataract surgeons must be increasingly interested in further improving the outcomes of cataract by measuring signs and symptoms of dry eye to improve the quality of vision after surgery and therefore the satisfaction of patients.

For these reasons, it should be mandatory to routinely introduce the ocular surface status examination before performing a phacoemulsification to prevent or promptly correct signs and symptoms of dryness before and after surgery itself. This preoperative examination could offer the opportunity to discuss with the patients the possibility of ocular dryness symptoms and signs and visual fluctuation after surgery, giving the occasion to control the patient’s concern for these ocular disorders and making the clinicians aware of dry eye symptoms and signs in an otherwise healthy eye [20].

Our study revealed that the visual acuity significantly improved after cataract extraction, as expected, and also the vision-related quality of life examined by the NEI-VFQ questionnaire generally improved after cataract extraction for general vision, reading and vision dependency, but the better vision-related quality of life was recorded for patients receiving artificial tears, demonstrating/suggesting the role of ocular surface regularity and not of surgery alone.

Our study therefore confirms the onset or worsening of ocular dryness after cataract extraction and the role of artificial tears in controlling signs and symptoms of dry eye but has the added value to be the first prospective study to evaluate usefulness of artificial tears prescribed not the day after surgery but 15 days later.

Our choice was determined by the experience of the poor adherence to prescribed therapy if therapeutic regimens are too complex. Literature is lacking regarding studies about ocular therapies and patients’ adherence apart from glaucoma, a chronic disease that requires use of topical eye drops in multicombinations more times a day. Two recent specific studies regarding adherence in glaucoma patients [21,22] pointed out that more complex therapeutic regimens are more associated with higher non-adherence [21] and that significant non-adherence correlates to higher dose frequency [22].

After cataract surgery, our patients were required to use two kinds of drops 4 times a day, meaning that patients were required to instill drops 8 times a day for 15 days. It is reasonable to believe that an additive prescription of another bottle (the third) of artificial tears to instill 3 times a day (for a total of 11 instillation a day) could become unprobeable. Our choice reached a significant reduction of dryness and a significant improvement in symptoms of ocular surface disease at the visit after 30 days (45 from surgery), and this was maintained until the last visit 90 days after surgery. The OSDI questionnaire statistically improved, not only from time of artificial tears instillation but also from preoperative data leading to normal value most patients using the artificial tears, while patients not using artificial tears worsened from mild to moderate values from baseline to 90 days visit.

As a final comment about the paper of Li et al. [2], the authors observed that patients whose cornea and conjunctiva fluorescein staining was positive 1 week after cataract surgery still had positive results 3 months after surgery: authors suggested that patients should be followed at least for 3 months after cataract surgery to study their ocular surface status. Our data confirm this suggestion since a worsening in symptoms as well as in BUT, corneal staining and conjunctival hyperaemia was noted in those patients not receiving artificial tears after cataract extraction when compared to baseline findings that are persistent over the time with a trend to worsening.

Our study presents pros and cons. The pros were that it was randomised, single blind and controlled.

The limits included the small sample size and the difficulty in assessing dry eye since there is no gold standard test.

Our study demonstrates that adding artificial tears 15 days after cataract surgery by phacoemulsification significantly reduces the signs (BUT, Schirmer, corneal staining, hyperaemia) and symptoms (OSDI and NEI VFQ 25 items) of dry eye experienced by the majority of operated patients avoiding the affect on adherence to steroid and antibiotic topical therapies that are fundamental and necessary/mandatory in the first/early post-surgery period.

*WHAT WAS KNOWN*

Cataract surgery causes the onset or the worsening of dry eye. Artificial tears use can reduce dry eye in these patients.

*WHAT THIS PAPER ADDS*

Dry eye after cataract extraction negatively impacts quality of vision and on vision-related quality of life: cataract surgeons must improve the outcomes of cataract by measuring signs and symptoms of dry eye pre- and post-operatively.

Artificial tears added 15 days after cataract surgery significantly reduce signs and symptoms of dry eye, while avoiding the affect on adherence to steroid and antibiotic topical therapies that are mandatory in the early post-surgery period.

## Figures and Tables

**Table 1:** Timetable of scheduled visits and examinations (pod: post-operative day).

Activity	Visit 1 T0 (Screening)	Visit 2 Tx (Surgery)	Visit 3 T1 (1 Pod)	Visit 4 T15 (15 Pod ± 3)	Visit 5 T45 (45 Pod ± 7)	Visit 6 T90 (90 Pod ± 15)
Informed consent	X					
Demographic and History	X					
Physical examination	X					
Ocular examination	X		X	X	X	X
Visual acuity	X			X	X	X
Eligibility criteria	X					
Surgery data			X			
Randomisation	X					
Eye drop prescription				X	X	X
Schirmer I	X			X	X	X
tBUT	X		X	X	X	X
Corneal fluorescein staining	X		X	X	X	X
OSDI	X			X	X	X
NEI-VFQ 25	X					X
Adverse events				X	X	X

**Table 2:** Clinical signs over time (SOC = standard of care; BUT = break up time; DES = dry eye syndrome = BUT < 10 sec plus Schirmer I < 10 mm; OSDI = ocular surface disease index). statistically significant values are on bold.

	Visit Time (Days)	SOC N = 16	Lacrimal Substitute (LS) N = 37	*p*-Value of Difference between Groups over Time
Visual acuity (decimals)	0	0.3 [0.2–0.4]	0.4 [0.2–0.4]	
	15	0.8 [0.6–1.0]	1.0 [0.8–1.0]	0.974
	45	0.9 [0.8–1.0]	1.0 [0.8–1.0]	0.999
	90	0.9 [0.8–1.0]	1.0 [1.0–1.0]	0.515
Schirmer I (mm)	0	10 [10–12]	10 [7–10]	---
	15	12 [8–16]	10.5 [6.5–21]	0.173
	45	10.5 [5–12.5]	12 [9–20]	**<0.001**
	90	10 [7–11.5]	12.5 [10–17]	**0.001**
BUT (s)	0	9 [7.5–10]	9 [8–10]	---
	15	10 [7–12]	10 [9–11.5]	0.455
	45	8 [6–10]	10 [8–12]	**0.009**
	90	7.5 [6–9.5]	9 [10.5–13]	**<0.001**
Corneal fluorescein staining (Grade > 0) Number (%)	0	0 (0)	5 (13.5)	
	15	2 (13.3)	1 (2.8)	0.981
	45	3 (18.7)	4 (11.8)	0.982
	90	3 (18.7)	2 (5.6)	0.981
Conjunctival hyperaemia (Grade > 0) Number (%)	0	0 (0)	4 (11.1)	
	15	1 (6.7)	6 (16.7)	0.990
	45	1 (6.7)	5 (15.1)	0.990
	90	2 (12.5)	5 (15.1)	0.990
DES Number (%)	0	5 (31.3)	15 (40.5)	
	15	6 (40)	16 (44.4)	0.979
	45	8 (50)	13 (38.2)	0.114
	90	8 (50)	9 (25)	**0.019**
OSDI score	0	21.3 [14.6–31.6]	18.3 [12.5–42.5]	
	15	17.4 [12.5–22.5]	12.2 [6.3–25]	0.096
	45	19.2 [14.7–23.4]	8.3 [5–22.2]	**0.026**
	90	24.1 [14–37.4]	7.5 [4.9–16.8]	**<0.001**

**Table 3:** NEI-VFQ 25 item: scores at baseline (0) and three months later 90 days. Statistically significant values are on bold.

NEI-VFQ Subscale Median [IQR]	Visit Time (Days)	SOC N = 16	Lacrimal Substitute (LS) N = 37	*p*-Value of Difference between Groups over Time
General health-GH	0	50 [25–50]	50 [50–50]	
	90	50 [25–62.5]	50 [50–50]	0.186
General vision-GV	0	40 [40–60]	40 [40–60]	
	90	60 [60–80]	80 [60–80]	**<0.00** **1**
Ocular pain-OP	0	81.2 [62.5–100]	75 [62.5–87.5]	
	90	87.5 [81.2–100]	87.5 [75–100]	0.708
Near activity-NA	0	83.3 [54.2–87.5]	66.6 [50–83.3]	
	90	91.6 [79.2–100]	83.3 [75–91.6]	**0.004**
Distance activity-DA	0	91.6 [70.8–100]	83.3 [58.3–91.6]	
	90	95.8 [79.2–100]	91.6 [83.3–100]	0.443
Vision-specific social functioning-VSSF	0	93.7 [87.5–100]	100 [75–100]	
	90	100 [87.5–100]	100 [87.5–100]	0.347
Vision-specific mental health-VSMH	0	90.6 [68.7–93.7]	75 [56.2–93.7]	
	90	90.6 [84.4–93.7]	87.5 [75–93.7]	0.075
Vision-specific role dependency-VSRD	0	87.5 [87.5–100]	75 [62.5–100]	
	90	100 [87.5–100]	100 [75–100]	0.404
Vision-specific dependency-VSD	0	100 [83.3–100]	100 [75–100]	
	90	100 [100–100]	100 [91.6–100]	**0.048**
Driving-D	0	81.2 [62.5–100]	81.2 [56.2–100]	
	90	81.2 [75–93.7]	87.5 [75–100]	0.645
Colour vision-CV	0	100 [100–100]	100 [75–100]	
	90	100 [100–100]	100 [100–100]	0.238
Peripheral vision-PV	0	100 [75–100]	75 [50–100]	
	90	100 [75–100]	100 [75–100]	0.776
Total mean	0	86.5 [72.5–96.6]	75.6 [63.6–85.2]	
	90	89.4 [82.5–93]	85.5 [79.8–92.6]	**0.030**

## Data Availability

Data are available from Authors.
